# Physician experiences implementing antimicrobial stewardship rounds in pediatric hospital medicine: An exploratory, qualitative study

**DOI:** 10.1017/ash.2021.175

**Published:** 2021-07-21

**Authors:** Megan L. McCreary, Alena Tse-Chang, Karen L. Forbes, Jessica L. Foulds

**Affiliations:** 1 Department of Medicine, Faculty of Medicine & Dentistry, University of Alberta, Edmonton, Alberta, Canada; 2 Department of Pediatrics, Faculty of Medicine & Dentistry, University of Alberta, Edmonton, Alberta, Canada; 3 Division of Pediatric Infectious Disease, Department of Pediatrics, Faculty of Medicine & Dentistry, University of Alberta, Edmonton, Alberta, Canada; 4 Division of Pediatric Hospital Medicine, Department of Pediatrics, Faculty of Medicine & Dentistry, University of Alberta, Edmonton, Alberta, Canada

## Abstract

**Objectives:**

An antimicrobial stewardship intervention was implemented for pediatric medicine units using an in-person rounds-based approach to provide stewardship recommendations and education from an antimicrobial stewardship physician and antimicrobial stewardship pharmacist.

**Design, Setting, Participants, and Methods:**

In this exploratory qualitative study, purposeful sampling was used to recruit participants for individual interviews at a tertiary- and quaternary-care referral center. Pediatricians and residents who attended ≥1 stewardship round were included. A semistructured interview guide was created focusing on perceptions of antimicrobial stewardship, personal experiences at stewardship rounds, and perceived impacts on patient care. Using a constant comparative analysis approach, codes were developed and collapsed into themes.

**Results:**

Overall, 8 pediatricians and 10 residents completed interviews. Qualitative analysis yielded 3 themes: insights into clinical reasoning, opportunity for growth and learning, and establishing and exploring professional relationships. The handshake-rounds approach encouraged participants to critically evaluate antimicrobial choices and to engage in discussion with the antimicrobial stewardship team. Participants felt validated at stewardship rounds and gained confidence prescribing antimicrobials. Face-to-face interaction reduced reluctance for some participants to consult infectious disease (ID) service; however, others worried that physicians may avoid ID consultation because of stewardship rounds.

**Conclusions:**

Participants found stewardship rounds to be an effective strategy for education and development of clinical reasoning skills for optimal antimicrobial prescribing—choosing wisely or choosing rightly. The effects of stewardship rounds on timing and frequency of ID consultation are interesting. Further research into important patient outcomes and consultation practices are needed locally, but our experiences may help others to reflect on the power of conversation and relationships in antimicrobial stewardship.

Antimicrobial stewardship programs aim to optimize antimicrobial use, improve patient outcomes, limit antimicrobial resistance, and reduce costs. Coordinated interventions promote selection of appropriate agents, dose, route, and therapy duration.^1^ These interventions include prospective audit with feedback (PAF), formulary restriction or preauthorization for certain agents, education, clinical practice guidelines, clinical decision support, and de-escalation of therapy.^1,2^ PAF and preauthorization are core strategies that many antimicrobial stewardship programs have employed,^1,3^ but PAF may have a greater impact on decreasing antibiotic use.^4^

Expanded from PAF, handshake stewardship emphasizes in-person rounding-based feedback delivery.^5^ Pharmacist–physician review of all antimicrobials is a key element of handshake stewardship, with no restriction or preauthorization. Initiated by Hurst et al^5^ in 2013, handshake stewardship was declared a leading practice in 2018 by The Joint Commission in the United States.^6^ The handshake stewardship approach yields high acceptance rates across many services, resulting in significantly decreased antimicrobial use.^5,7^ After 5 years of handshake stewardship, MacBrayne et al^8^ reported 25% reduction in antimicrobial use, demonstrating sustainable efficacy of handshake stewardship. Variations in handshake stewardship exist in numerous centers, targeting different services,^9–11^ or entire hospitals.^12,13^

Anecdotally, the success of handshake stewardship lies in the ongoing face-to-face interaction between the antimicrobial stewardship program and medical teams, fostering collaboration and promoting real-time education about optimal antimicrobial management.^5,7,10,14^ Our center implemented a modified form of handshake stewardship in 2018 in our Pediatric Medicine units as an active patient-care intervention,^15^ involving postpresciption review and feedback. We sought to qualitatively explore this strategy by interviewing participating pediatricians and pediatric residents one year after implementation.

## Methods

Our children’s hospital is a tertiary- and quaternary-care facility with 2 large pediatric medicine admitting teams. With a daytime in-house attending physician, 3–8 trainees per team practice family-centered rounds. Antimicrobial prescribing is not restricted, and no preauthorization of antimicrobials is mandated. Our antimicrobial stewardship service consists of 3 antimicrobial stewardship physicians (pediatric infectious disease [ID] specialists) and 2 ID and antimicrobial stewardship pharmacists who rotate weekly. Stewardship rounds are based on a handshake stewardship design. Twice weekly, all new antimicrobial prescriptions for pediatric medicine inpatients are reviewed by the antimicrobial stewardship team (1 antimicrobial stewardship physician and 1 antimicrobial stewardship pharmacist). All applicable patients are discussed during in-person antimicrobial stewardship rounds between the antimicrobial stewardship team and pediatric medicine teams. Antimicrobial stewardship team recommendations are classified as continue, discontinue, change, or optimize antimicrobial prescription. Other recommendations include education, additional investigations, or consult the ID service. Even if there are no specific recommendations, the antimicrobial stewardship team meets with pediatric medicine teams to discuss questions.

Purposeful sampling was used to recruit participants for individual interviews. Attending inpatient pediatricians and pediatric residents who attended at least 1 stewardship round were included. Participants were not offered incentive to participate. This study was approved by our institutional research ethics board.

A semistructured interview guide was created by an antimicrobial stewardship physician (A.T.C.) and hospitalist pediatricians with qualitative methodology experience (K.L.F. and J.L.F.), focusing on perceptions of antimicrobial stewardship, personal experiences with stewardship rounds, and perceived patient-care effects. An interview guide is available in Appendix online. A medical student (M.L.M.) conducted all interviews. Interviews were digitally recorded and transcribed verbatim. Pseudonyms were used and identifying information was removed during transcription. Interviews were conducted until saturation was reached.

Transcripts were analyzed after each interview using constant comparative analysis, which allowed for identification of additional topics to explore in future interviews. A researcher coded the first interview to build a coding framework. The same researcher then coded subsequent interviews, adding new codes to the framework as they were identified. Using this framework, coded data were analyzed by 4 researchers and were collapsed into subthemes then themes.

## Results

**Fig. 1.**
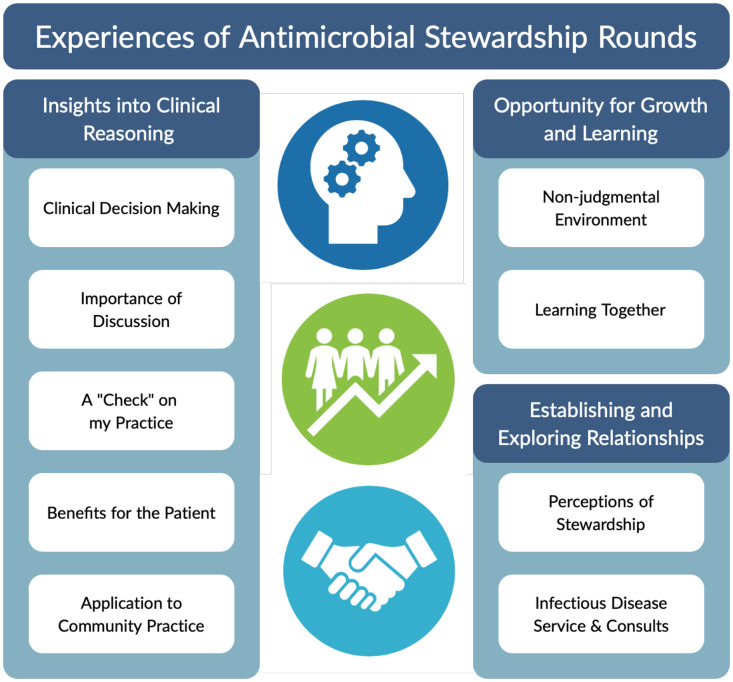
Experiences of antimicrobial stewardship rounds. Analysis of individual interviews revealed 3 themes and 9 subthemes related to the experiences of pediatricians and residents at stewardship rounds. These themes highlight the importance of clinical reasoning, personal growth and learning, and exploring professional relationships.

In total, 8 pediatricians, 4 junior residents, and 6 senior residents completed interviews. The mean number of years of hospitalist practice for pediatricians was 14.3 years (SD, 10.6). The mean number of stewardship rounds attended by participants was 8.1 rounds (SD, 7.1). Overall, 41 codes were identified, collapsed into 9 subthemes then 3 themes: insights into clinical reasoning, opportunity for growth and learning, and establishing and exploring professional relationships (Fig. 1).

### Insights into clinical reasoning

Finding a balance between effective use of resources and preventing poor patient outcomes was challenging for participants, and some felt they previously used antimicrobials as a “safety net.” Stewardship rounds encouraged participants to critically evaluate antimicrobial choices because they later discussed decisions with the antimicrobial stewardship team. Furthermore, participants felt validated at stewardship rounds because the antimicrobial stewardship team often reaffirmed their choices. Some described increased confidence in their ability to prescribe antimicrobials for future patients.

*“It’s nice to get that little boost of confidence to say like this probably doesn’t need antibiotics so it’s okay to stop them. So, it makes me aware of it, makes me think about it more often and also reinforces what I think I should do but may be a bit nervous to do.” (R-02)*

*“I’ve found that usually we’ll make a decision on rounds…then when we discuss it in [stewardship] rounds, it’s nice to have that validated or agreed upon with the [AS] team as well…we’re actually pretty much on the same page.” (P-03)*

Stewardship rounds provided participants an opportunity to meet with antimicrobial stewardship physicians in an informal, conversational atmosphere. Participants appreciated how this approach allowed them to explain their reasoning and engage in discussion with the antimicrobial stewardship team. This approach was particularly helpful for complex patients for whom there may be no clear answer based on guidelines. Most participants had not experienced a situation in which they disagreed with antimicrobial stewardship team recommendations. Of those who had a disagreement, they described it as collegial and felt supported by the antimicrobial stewardship team.

*“A lot of the cases that we discuss during antimicrobial stewardship [rounds] aren’t like the black and white cases where you know exactly what to do and we have good evidence for. The cases that get discussed are the ones where there isn’t that clear-cut evidence so we can actually bounce ideas off each other. Not only do you have the infectious disease [AS] specialist and the [AS] pharmacist, you’ve also got the other pharmacist on the teams as well as all the different staff pediatricians and senior residents so there’s a lot of people in the room that can help you make those decisions.” (R-04)*

Participants found it reassuring that the antimicrobial stewardship team reviewed their patients in case something was missed or could be improved. Stewardship rounds also provided an opportunity for participants to review their patients and ensure decisions were evidence-based.

*“I think like sometimes we, for the patients that are on antibiotics, we don’t think about their antibiotic choices unless we hit an obstacle or a challenge…then we will consult ID. So, I like how the antimicrobial stewardship program does a review of all the patients on antibiotics versus having to consult ID which is on a case-by-case basis where there might be some patients that are missed.” (R-07)*

Participants explained how patients are the ones who ultimately benefit from stewardship rounds. A common example was how the antimicrobial stewardship team encouraged them to put stop dates in place.

*“…Obviously our main concern is patient care, so you want to be doing the right thing and here you have the opportunity to have a consultant who is directing that. So, you know the patient gains right, both in terms of the number of antibiotics we use, the narrower the spectrum, decreasing resistance, overall patient care, but also when we transition from intravenous to oral more quickly and getting the patient out.” (P-07)*

### Opportunity for growth and learning

Participants described the stewardship-round environment as supportive and nonjudgmental, and felt they could engage in discussion without fear of criticism. Some participants knew the antimicrobial stewardship physicians prior to stewardship rounds and felt comfortable working with them in this context. None of the pediatricians worried about how others perceived them at stewardship rounds. Some residents expressed concern about this but appreciated that it made them critically evaluate their decisions.

*“I think it’s just having more of a discussion. It’s not just ‘this is what we do’, it’s more of ‘well this is what we do and why, and this is why maybe your choice is not the best’. Having an open dialogue makes it less intimidating. And I think approaching it as more of a learning experience rather than an audit per se makes it helpful.” (R-05)*

Senior residents described the importance of the educational component. Some felt that junior residents and medical students should attend stewardship rounds because of this. Participants appreciated having other pediatric medicine teams present for stewardship rounds because they learned from the other team’s cases when a brief backstory was given for each patient.

*“I think that they’re a great way for us, as residents, to learn why we make certain antibiotic choices and when to stop antibiotics. And we’re able to ask questions directly to the [AS] pharmacists and hear what they are doing on the other team, not just your own team.” (R-06)*

### Establishing and exploring professional relationships

All participants viewed antimicrobial stewardship as a positive and necessary aspect of ID medicine. Despite perceived importance, some felt that antimicrobial stewardship is easily forgotten so having stewardship rounds emphasized its value. Participants noticed a shift in culture as physicians became more accepting of antimicrobial stewardship over time. However, they worried that some physicians might resist antimicrobial stewardship if they felt that their autonomous decision making were threatened. Participants also described benefits of this intervention to the healthcare system in terms of decreasing cost and antimicrobial resistance.

*“I think people are quite aware of the importance, but I think adding the rounds definitely keeps it in the forefront like ‘are we doing it, are we choosing the best antibiotics.’ So I think in general people realize it’s important but having the rounds just kind of adds that extra emphasis.” (P-05)*

*“I think for the most part [AS] is very well received. I think that people are really understanding that it’s a needed aspect of infectious diseases. I think that some people maybe do perceive that it is interfering with their own decision making as the [Most Responsible Physician], but I don’t think that’s a common perception, I think it’s just a few people.” (R-03)*

Stewardship rounds provided face-to-face interaction with antimicrobial stewardship physicians. Thus, some participants felt validated pursuing an ID service consult. In contrast, others worried that physicians may avoid ID consults and instead wait for input at stewardship rounds, specifically for simple questions on duration or dosing. Participants found it challenging to have both the ID service and the antimicrobial stewardship team involved in a patient’s care, particularly when other services were also involved.

*“I think something that has been very helpful is for residents that haven’t gone through an ID rotation yet or haven’t had a lot of interaction with the ID staff because it really helps with that sort of face-to-face when you just need to ask them a quick question passing by or even being able to call them on-call overnight…having a little bit of face-to-face and building those relationships has been very helpful.” (R-01)*

## Discussion

This qualitative study explored physician experiences and perceptions of an inpatient pediatric medicine handshake stewardship program 1 year following implementation. Interviews of attending and resident physicians allowed rich exploration of experiences previously not as deeply described in the literature because studies to date have primarily focused on survey data.^16^ Our handshake stewardship program was well received, and it highlighted the importance of stewardship, continuous quality improvement, and patient safety. The positive experiences thus far suggest that this model of PAF can be expanded to other services at our center.

Effective faculty development initiatives include those with collaborative and consultative approaches; those utilizing trusted peer contributors; and those that consider workplace relationships, context, and culture with concern of contributors being addressed and supported.^17^ With multilevel learners being part of stewardship rounds, the educational component and real-time feedback on clinical decisions did have some physicians worried about how their decision making may be viewed by others. However, having multiple inpatient teams present at stewardship rounds and reviewing cases not solely under the care of one attending physician was highlighted as an opportunity to mutually learn.

Few participants in our study experienced disagreements with the antimicrobial stewardship team. For those who did, they felt the disagreement to be collegial. Prior studies have identified predictors of pediatric antimicrobial stewardship recommendation disagreements that included specific patient-level, programmatic, and provider-level factors.^18,19^ For each year following completion of residency, training providers were 2.4% less likely to follow antimicrobial stewardship recommendations at a single US pediatric hospital.^18^ In our study, pediatrician experience was diverse, with hospitalist practice ranging from 2.5 to 38 years, although this did not seem to influence antimicrobial stewardship recommendation disagreements or perceptions of prescribing autonomy. Regardless of use of restrictive or persuasive strategies, how the antimicrobial stewardship team communicates with end-user prescribers and the prescribers’ experiences are essential to the sustainability and success of these programs.

Our participants described stewardship rounds as encouraging when discussing complex cases with the pediatric ID service and provided validation in pursuing ID consults. Physicians worried, however, that fewer ID consults in general may be pursued; worry not stemming from the straightforward cases now managed more comfortably but due to lack of role clarity between the antimicrobial stewardship team and ID service. Following implementation of their stewardship rounds, Messacar et al^14^ noted that their pediatric ID consultations increased by 57% (35% when standardized per 1,000 admissions). Local data collection and analysis will serve as an important next step in evaluating our program outcomes, surveillance, and balancing measures.

One year after the implementation of stewardship rounds, participants continued to enjoy engaging in discussion with the antimicrobial stewardship team. How these experiences change over time, when novelty wears off, will be important to monitor. Fears of threatened autonomy or clinical decision making could increase over time. This trend has not been identified by prescribers surveyed in an antimicrobial stewardship program established for 15 years,^20^ but it will be an important experience to consider in our unique clinical context.

Although many insights were gained during our exploration of physician experiences of stewardship rounds, our study has limitations. Some participants had only participated in stewardship rounds once or twice, limiting longitudinal interactions with the antimicrobial stewardship team and possibly limiting diversity of experiences. Some physicians had baseline collegial relationships with members of the antimicrobial stewardship team, which may have influenced their experiences at stewardship rounds. Other limitations include those of qualitative methodology in general, in that exploration of differences in experiences based on level of training is not possible. We can use the subsequent percentage of agreed-upon recommendations and analyses of conditions for further work on low-value care opportunities for quality improvement.

In conclusion, prescriber perceptions and experiences are essential to consider when addressing change management. This qualitative study is the first to explore pediatrician and resident experiences with antimicrobial stewardship, particularly handshake stewardship. Our results highlight personal development, learning and leveraging growth mindsets for prescribers, how we connect and explore relationships, and the importance of fostering and supporting clinical decision making. Although these themes are context and participant specific, the lessons learned may be applicable to other antimicrobial stewardship programs.
